# Data regarding the experimental findings compared with CALPHAD calculations of the AlMo_0.5_NbTa_0.5_TiZr refractory high entropy superalloy

**DOI:** 10.1016/j.dib.2022.108858

**Published:** 2022-12-24

**Authors:** Patricia Suárez Ocaño, Leonardo Agudo Jácome, Inmaculada Lopez-Galilea, Reza Darvishi Kamachali, Suzana G. Fries

**Affiliations:** a Department for Materials Engineering, Bundesanstalt für Materialforschung und -prüfung (BAM), Unter den Eichen 87, Berlin 12205, Germany; b Institute for Materials, Ruhr-University Bochum, Universitätsstr. 150, Bochum 44801, Germany; c Materials Research Department (MRD), Ruhr-University Bochum, Universitätsstr. 150, Bochum 44801, Germany

**Keywords:** Transmission electron microscopy, Scanning electron microscopy, Microstructural characterization, Refractory high entropy alloys

## Abstract

This contribution contains the raw data used to compare experimental results with thermodynamic calculations using the CALPHAD method, which is related to the research article “The AlMo_0.5_NbTa_0.5_TiZr refractory high entropy superalloy: experimental findings and comparison with calculations using the CALPHAD method” [1], and therefore this article can be used as a basis for interpreting the data contained therein.

The AlMo_0.5_NbTa_0.5_TiZr refractory superalloy was characterized in the cast and annealed condition (1400 °C for 24 h) in order to measure grain size and to identify and measure the size and area fraction of the phases present.

The raw data of this article include X-ray diffraction (XRD) measurements, microstructural characterization by scanning and transmission electron microscopy (SEM and TEM), and elemental analysis by energy dispersive X-ray spectroscopy (EDX). XRD includes the determination of phases and the lattice parameters (A2, B2, and hexagonal structure). Microstructural analysis by scanning and transmission electron microscopy includes (1) identification of composition, size, and volume fraction of the present phases and (2) determination of grain size. Based on these experimental data, it is possible to identify similarities and discrepancies with the data calculated using the CALPHAD method for the alloy under study in Ref. [1], which provides the basis for better and more efficient development of reliable databases.

**Specifications Table**

**Table:** 

Subject	Material Science
Specific subject area	Microstructural characterization of a refractory high entropy superalloy
Type of data	Tables Images (SEM-BSE, TEM) Excel Files (XRD)
How the data were acquired	XRD: Seifert XRD 3000 PTS diffractometer SEM: FEI Quanta 3D ESEM SEM-EDX: EDAX Octane Elect SDDs TEM: JEOL JEM-2200FS TEM-EDX: Jeol JED-23000BU Si (Li) semiconductor with ultrathin organic/Al window. ImageJ and FIJI: Image Analysis Software.
Data format	Measured raw (XRD patterns, EDX analysis of the phases). Analyzed (lattice parameters, indexation of diffraction patterns in TEM, average grain sizes, thickness of bcc (A2) plates, B2 channels, and area fraction of B2 and Al-Zr-rich phase. Area fraction of the amorphous phase in the Al-Zr-rich phase.
Description of data collection	The XRD patterns were acquired using Seifert XRD 3000 PTS diffractometer operating with a Co-Kα radiation source (λ = 1.7902 Å). The SEM images were acquired using an FEI Quanta 3D ESEM integrated with an EDX EDAX Octane Elect SDDs detector: using an acceleration voltage between 20 kV and 30 kV and working distances between 5 and 10 mm. Metallographic samples were prepared by grinding and polishing and used for collecting the SEM-EDX and XRD data. The TEM images and TEM-EDX data were acquired using a JEOL JEM-2200FS TEM and a JED-23000BU EDX detector for the elemental analysis, respectively. The images were taken in conventional dark field (CTEM-DF) and scanning high angle annular dark field (STEM-HAADF) mode. TEM foils were prepared by electropolishing to collect the TEM and TEM-EDX data.
Data source location	Institution: Bundesanstalt für Materialforschung und -prüfung (BAM) City/Town/Region: Berlin Country: Germany Institution: Institute for Materials and Center for Interface-Dominated High-Performance Materials (ZGH), Ruhr-Universität Bochum. City/Town/Region: Bochum/ North Rhine-Westphalia (NRW) Country: Germany
Data accessibility	Repository name: Mendeley Data Direct URL to data: https://data.mendeley.com/datasets/d742ccty5f/4
Related research article	P. Suárez Ocaño, S.G. Fries, I. Lopez-Galilea, R. Darvishi Kamachali, J. Roik, L. Agudo Jácome, The AlMo_0.5_NbTa_0.5_TiZr refractory high entropy superalloy: Experimental findings and comparison with calculations using the CALPHAD method. Mater. Des. 217 (2022) 110,593. https://doi.org/10.1016/j.matdes.2022.110593

**Value of the Data**

• The data presented in this article includes all the raw data and processing for the AlMo_0.5_NbTa_0.5_TiZr refractory high entropy superalloy in cast and annealed states reported in the related article (Ref. [1]), which are useful for determining grain size, identification/area fraction of the phase structures and their volume fraction, being important for general alloy characterization.
• The procedures used in the determination of grain size, identification of phases, and their volume fraction could be useful for other researchers interested in determining these parameters in any type of alloy. These data may be useful to scientists and researchers in the high entropy alloy community, a field that is constantly evolving.
• The compilation of these data (BSE microphotographs, XRD patterns, TEM images and EDX spectra, grain size, and area fraction tables) can be used to develop image analysis algorithms to improve computer-aided analysis of microstructures.
• A method for simulating XRD patterns is provided, which can be used in the study of these types of relatively new alloys for which there are few XRD databases. In addition, the composition and structure of each phase in this alloy could be implemented in alloy design software.

## 1 Data Description

The XRD data presented in this article are expressed as 2*θ* versus intensity plots obtained for the alloy under study (AlMo_0.5_NbTa_0.5_TiZr refractory superalloy, RSA). The raw XRD data are provided as Excel files (see XRD folder in the dataset), and a summary of the recorded diffraction patterns is shown in Fig. 8 of Ref. [1]. The patterns simulated with PowderCell software [2] are included in the dataset (.cel files) for the different phases in the cast (AC) and annealed (AN, 1400 °C for 24 h) specimens. In both cases, most of the diffraction peaks could be indexed according to body-centered cubic, bcc structures (A2, space group $Im\bar{3}m$ and B2, space group $Pm\bar{3}m$). Besides the bcc-based peaks, additional peaks, corresponding to the Al-Zr-rich hexagonal intermetallic (space group *P6_3_/mcm*), were detected.

Table 1 summarizes the crystal structure of the phases, their space group, and the lattice parameters obtained by simulating the different phases in both states (AC and AN).

**Table 1:** Summary of the different phases found in the samples studied (AC and AN) with their respective space group and lattice parameters.

			Lattice parameters (Å)		
State	Phase	Space group	*a*	*b*	*c*
As-cast (AC)	B2	$Pm\bar{3}m$	3.32	3.32	3.32
	A2	$Im\bar{3}m$	3.28	3.28	3.28
	Hexagonal intermetallic	*P6_3_/mcm*	8.31	8.31	5.52

Annealed (AN, 1400 °C for 24 h)	B2	$Pm\bar{3}m$	3.31	3.31	3.31
	A2	$Im\bar{3}m$	3.27	3.27	3.27
	Hexagonal intermetallic	*P6_3_/mcm*	8.31	8.31	5.52

**Fig. 1:**
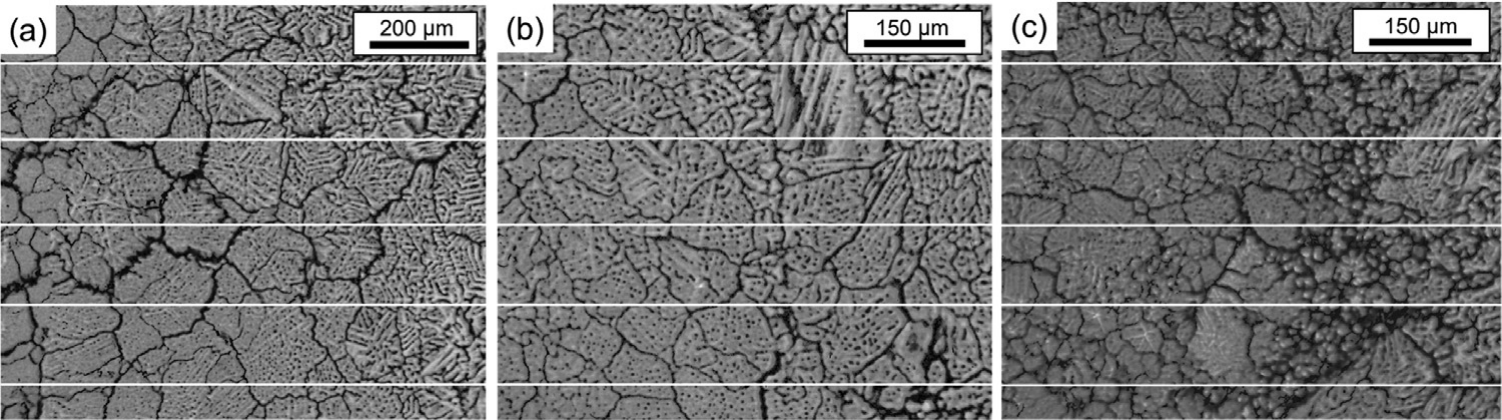
Images for grain size determination in the alloy AC, with the lines drawn to measure the number of intersections with the grain boundaries, (a) 300x and (b, c) 400x.

**Fig. 2:**
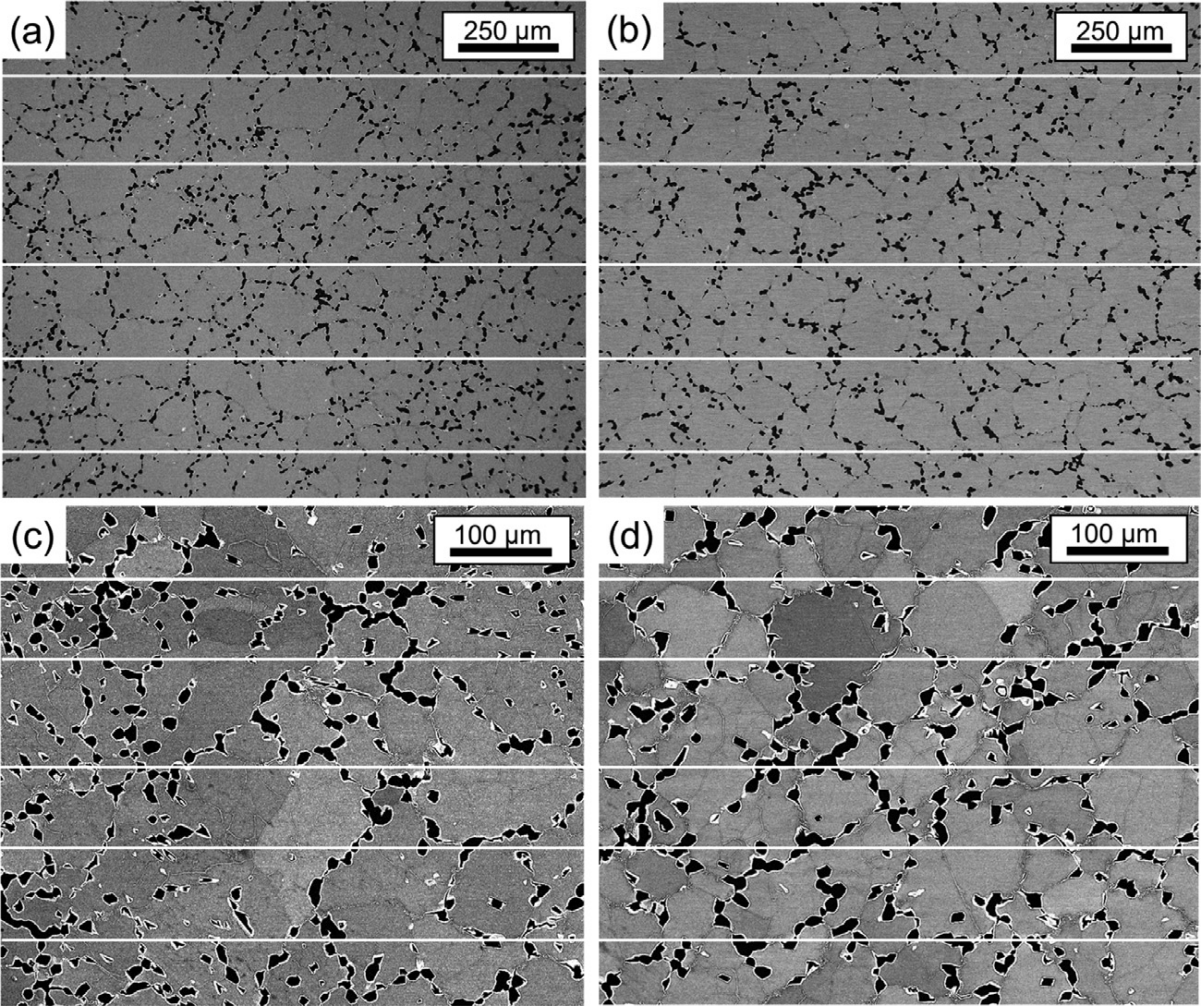
Images for grain size determination in the alloy AN, with the lines drawn to measure the number of intersections with the grain boundaries, (a, b) 200x and (c, d) 500x.

The microstructures of the AC and AN were analyzed by scanning electron microscopy (SEM) equipped with a backscatter electron (BSE) detector. BSE micrographs taken at low and medium magnification are presented to document the grain size “*d”* in the two different states (see Figs. 1 and 2 of Section 2). The average grain size for both states is given in Table 2.

**Table 2:** Average grain size *d* of the RSA in the AC and AN state. The uncertainty is given by the standard deviation.

State	Diameter average *d* (µm)
As-cast (AC)	78.3 ± 19.9
Annealed (AN, 1400 °C for 24 h)	77.8 ± 8.6

Table 3 summarizes the average area fractions of the Al-Zr and B2 phases in AC and AN. In the AC alloy, the nanostructure consisted of plate-like precipitates with ≈ 2–20 nm thickness embedded in channels with a thickness of ≈ 2–10 nm. The alloy AN exhibited a heterogeneous nanostructure having plate-like precipitates with a thickness of ≈ 10–100 nm embedded in a continuous phase with a thickness of ≈ 3–30 nm. The measurements can be found as Excel files in the dataset (channels.cvs and precipitates.cvs), as well as the images used for the determination.

**Table 3:** Average area fraction of the Al-Zr-rich and B2 phases determined with ImageJ software. The uncertainty is given by the standard deviation.

State	Area fraction of the Al-Zr-rich phase (%)	Area fraction of the B2 phase (%)
As-cast (AC)	8 ± 4	39 ± 4
Annealed (AN, 1400 °C for 24 h)	13 ± 6	37 ± 9

Since the Al-Zr-rich phase in the AC state is composed of amorphous and crystalline fractions, a method was implemented to determine the crystalline fraction within the analyzed regions (see Section 2). The AC sample analyzed by TEM yielded only 5 ± 4% of the Al-Zr-rich regions as crystalline as reported in Ref. [1]. The local chemical compositions of the phases measured at the three different phases in SEM- and TEM-EDX for the studied alloy are given in Table 2 of Ref. [1] and the raw data can be found in the dataset (EDX folder). A table with the name, format, and a brief description of each dataset file contained in Mendeley, is provided at the end of this work.

## 2 Experimental Design, Materials and Methods

XRD analysis were conducted for the investigated alloy using a Seifert PTS 3000 diffractometer operating with a Co Kα radiation source (λ_Kα1_ = 1.7902 Å). The patterns were acquired using a scattering range of 10–100° for the as-cast state (AC) and 10–120° for the annealed (AN) state with a step size of 0.05° and Bragg-Brentano geometry. To obtain the lattice parameters listed in

Table 1, patterns of the identified phases were simulated using the PowderCell software [2], and the crystal files were manually edited considering the present elements of each phase, the space-group number, the position of the atomic species, and the occupancy of the specific coordinates of the lattice. The lattice parameters were then modified until the exact positions of the peaks in the experimental pattern were reached. Table 4 lists all the parameters chosen to determine the simulated patterns for the lattice parameter identification. Finally, the identification was performed by comparing the experimental and simulated patterns.

**Table 4:** Parameters used for the simulation of the present phases in the studied alloy.

						Atom position		
Phase	Space group	Space group number	Lattice parameter (Å)	Elements	Wyck	X	Y	Z
B2	$Pm\bar{3}m$	221	*a* *=* *b* *=* *c* *=* *3.32*	Al	1a	0	0	0
				Ti	1a	0	0	0
				Zr	1b	0.5	0.5	0.5

A2	$Im\bar{3}m$	229	*a* *=* *b* *=* *c* *=* *3.28*	Mo	2a	0	0	0
				Nb	2a	0	0	0
				Ta	2a	0	0	0

Hexagonal intermetallic (Al-Zr-rich)	P6_3_/mcm	193	*a* *=* *b* *=* *8.31 c* = 5.52	Al	6 g	0.62	0	0.25
				Zr	4d	0.33	0.66	0

Samples for scanning electron microscopy were prepared by conventional metallography with emery paper (SiC) grades p320, p600, p1200, p2500, and p4000 and polished with a silica solution of 50 nm particle size. Backscattered electron (BSE) images were acquired using a Quanta 3D scanning electron microscope (SEM, FEI Company) with an accelerating voltage of 20–30 kV and a working distance of ≈ 5–10 mm. Grain size (*d*) was determined using the Heyn linear intercept method described in ASTM E112 standard [4], using four images for each condition. Five lines were drawn in each image, as shown in Figs. 1 and 2 for AC and AN, respectively, to measure the number of intersections of each line with the grain boundaries. The average grain diameter (size) is then given by *d* = line length/number of intersections. 221 intersections were counted for the AC and 270 for the AN. Tables 5 and 6 summarize the parameters and average grain diameter *d* (used to determine the average grain size in Table 2) in the AC and AN states, respectively, for each line drawn in the four images in Figs. 1 and 2.

**Table 5:** Parameters and average grain size *“d”* in the AC state for each drawn line in the four images of Figs. 1 and 2a of Ref. [1]. The uncertainty is given by the standard deviation of the average grain size of each BSE image.

Figure	Line N°	N° intersections	Line length (µm)	*d* (µm)
Fig. 1a	1	13	993.8	76.4
	2	13	993.8	76.4
	3	13	993.8	76.4
	4	12	993.8	82.8
	5	15	993.8	66.3
			Average	75.7 ± 5.9

	Line N°	N° intersections	Line length (µm)	*d* (µm)

Fig. 1b	1	13	750.0	57.7
	2	12	750.0	62.5
	3	12	750.0	62.5
	4	12	750.0	62.5
	5	12	750.0	62.5
			Average	61.5 ± 2.2

	Line N°	N° intersections	Line length (µm)	*d* (µm)

Fig. 1c	1	16	750.0	46.9
	2	14	750.0	53.6
	3	8	750.0	93.8
	4	9	750.0	83.3
	5	11	750.0	68.2
			Average	69.14 ± 19.7

	Line N°	N° intersections	Line length (µm)	*d* (µm)

Fig. 2a of Ref. [1]	1	6	750.0	125.0
	2	6	750.0	125.0
	3	8	750.0	93.8
	4	7	750.0	107.1
	5	9	750.0	83.3
			Average	69.14 ± 19.7

**Table 6:** Parameters and average grain size “*d*” in the AN state for each drawn line in the four images shown in Fig. 2. The uncertainty is given by the standard deviation of the average grain size of each BSE image.

Figure	Line N°	N° intersections	Line length (µm)	*d* (µm)
Fig. 2a	1	18	1506.9	83.7
	2	18	1506.9	83.7
	3	17	1506.9	88.6
	4	20	1506.9	75.3
	5	19	1506.9	79.3
			Average	82.1 ± 5.0

	Line N°	N° intersections	Line length (µm)	*d* (µm)

Fig. 2b	1	17	1494.6	87.9
	2	16	1494.6	93.4
	3	17	1494.6	87.9
	4	17	1494.6	87.9
	5	17	1494.6	87.9
			Average	89.0 ± 2.5

	Line N°	N° intersections	Line length (µm)	*d* (µm)

Fig. 2c	1	8	589.2	73.6
	2	8	589.2	73.6
	3	9.5	589.2	62.0
	4	6.5	589.2	90.6
	5	8	589.2	73.6
			Average	74.7 ± 10.2

	Line N°	N° intersections	Line length (µm)	*d* (µm)

Fig. 2d	1	10.5	746.2	71.1
	2	10.5	746.2	71.1
	3	10	746.2	74.6
	4	11	746.2	67.8
	5	12	746.2	62.2
			Average	69.4 ± 4.7

The specimens for TEM were cut into slices of ≈ 500 µm thickness and ground to a thickness of ≈ 100 µm on both sides with SiC emery paper of grades p320, p600, and p1200. Then, they were punched out into 3 mm diameter disks for electropolishing in a twin-jet Struers Tenupol-3, using an electrolyte consisting of 950 ml ethanol (86.4%), 100 ml butanol (9.1%), and 50 ml perchloric acid (4.5%) at -30 °C, 30 V and 3.1 flow rate.

Transmission electron microscopy images were acquired using a TEM JEOL JEM-2200FS with a field emission gun (FEG), operating at an accelerating voltage of 200 kV. BF and DF CTEM images with their respective selected area diffraction patterns (SADPs) were acquired to identify the phases, in addition to STEM-HAADF and BF images to determine the volume fraction. For the alloy AC, CTEM-DF micrographs and their respective SADPs were taken in the interdendritic zone.

**Fig. 3:**
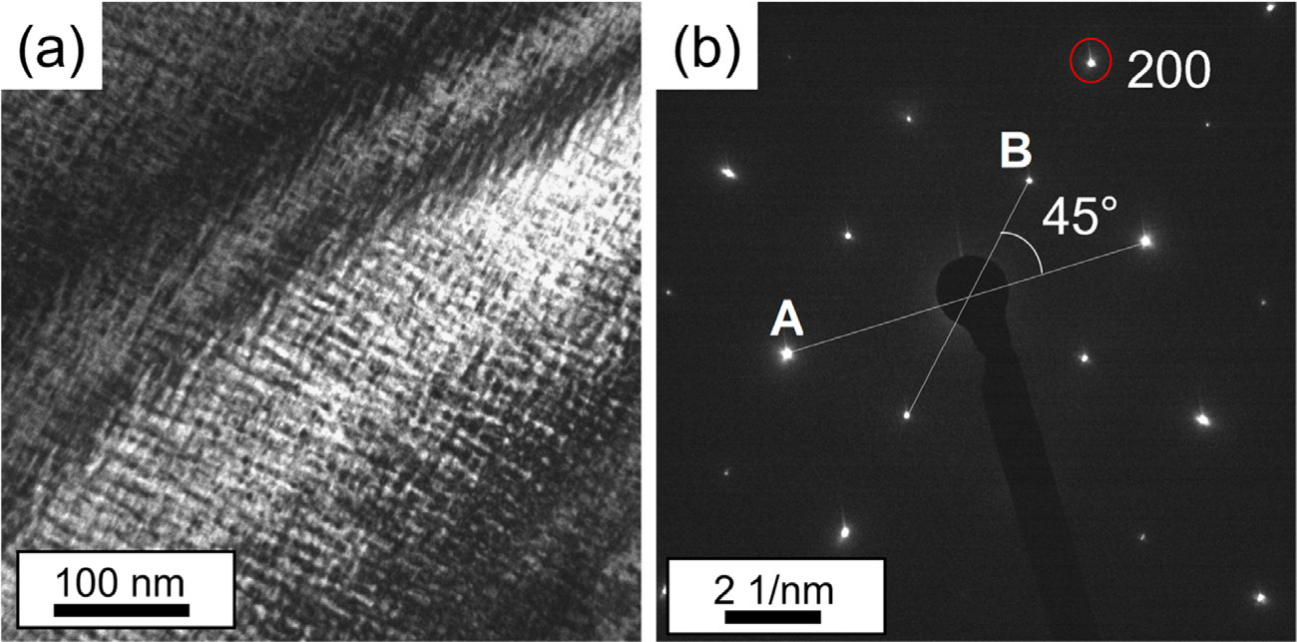
(a) CTEM-BF micrograph of the interdendritic region in AC (CTEM bright field image of the Fig. 5b from Ref. [1]). (b) SADP from (a) obtained along the [001] zone axis, showing the angle between “A” and “B” (modified version of Fig. 5c from Ref. [1]).

Fig. 3 shows a CTEM-BF micrograph of the interdendritic region in AC (a), with the respective SADP along the [001] zone axis (b) used to identify the phases in the interdendritic region. The SADPs used to characterize the bcc-based structures (A2 and B2) were indexed by measuring the lengths of two diffraction vectors and the angle formed by them, and the ratio A/B was compared with the diffraction patterns described in Appendix 4 – Ref. [5] for bcc alloys. The lines labeled “A” and “B” in Fig. 3b were measured, and the ratio A/*B* = 1.414, with a relative angle of 45° corresponds to bcc oriented along the [001] zone axis, as shown in Fig. 3b.

For the Al-Zr-rich phase region, the SADPs were indexed using the Java version of the electron microscopy suite (JEMS) [6], (Al3Zr5: ICDD- 04-003-0819, Al4Zr5: ICDD-00-048-1382, see zip file). The inset in Fig. 6c of Ref. [1] shows a simulated diffraction pattern using the ICDD crystal file 00-048-1382.

**Fig. 4:**
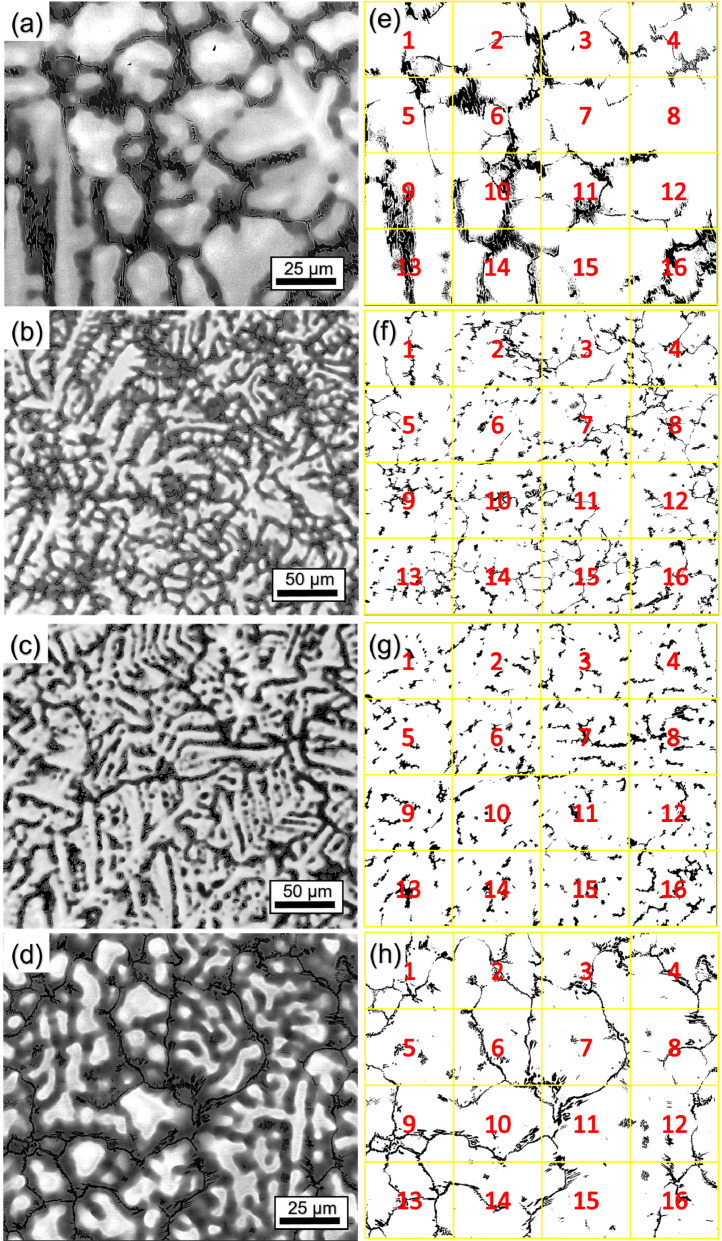
BSE micrographs of AC alloy taken at (a) 2000x, (b) 1000x and (c) 1000x (d) 2000x magnification. (e–h) Binarized images of (a–d), respectively. The black phase shown in (e–h) represents the Al-Zr-rich phase. The binarized images were divided into 16 images, as shown by the yellow squares and red numbers.

**Fig. 5:**
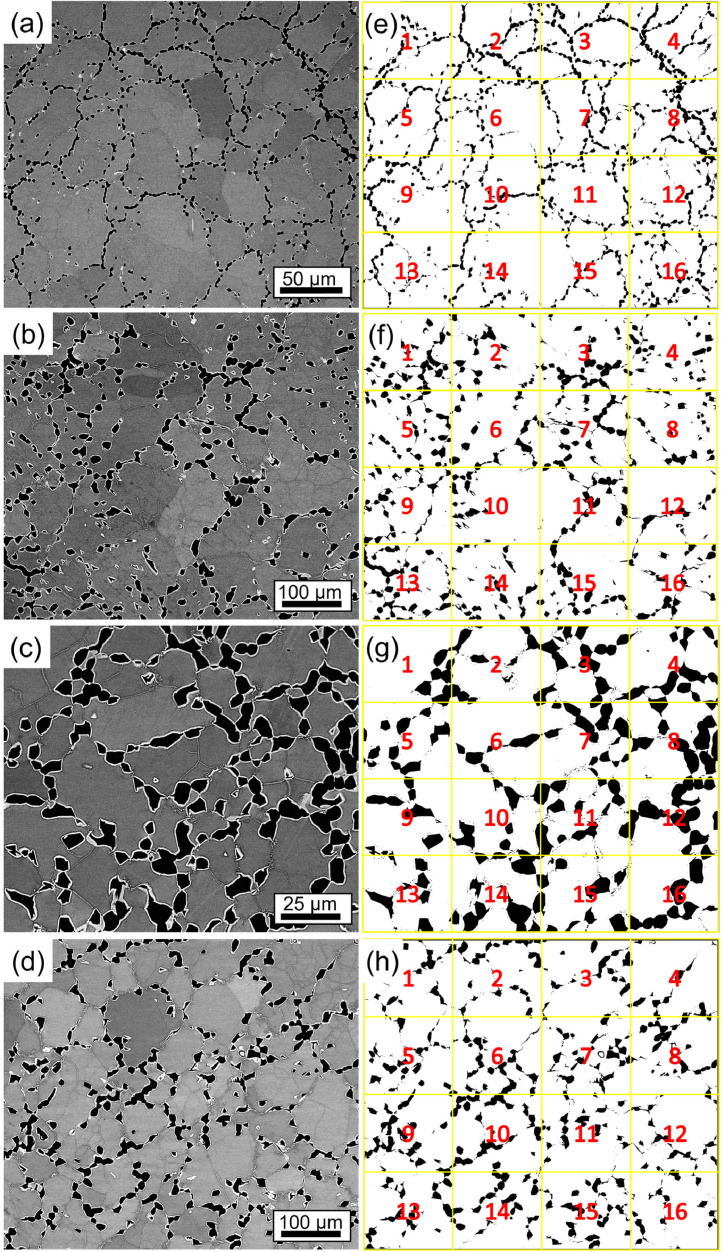
BSE micrographs of the AN alloy taken at (a) 260x and (b) 500x (c) 1000x (d) 500x magnification. (e–h) Binarized images of (a–d), respectively. The black phase shown in (e–h) represents the Al-Zr-rich phase. The binarized images were divided into 16 images, as shown by the yellow squares and red numbers.

**Fig. 6:**
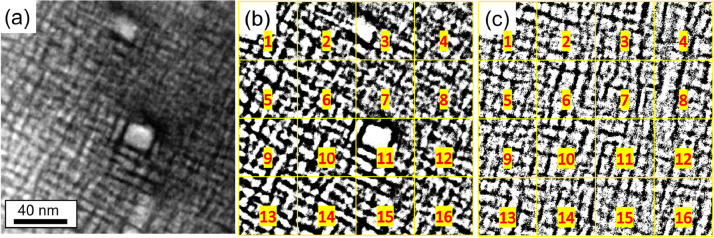
(a) STEM-HAADF micrograph of the AC alloy used to determine the area fraction of A2/B2 phases (800kx). (b) Binarized image of (a). (c) Binarized image of Fig. 5d in Ref. [1]. The black phase shown in (b) and (c) represents the B2 phase (channels). The binarized images were divided into 16 images, as shown by the yellow squares and red numbers.

**Fig. 7:**
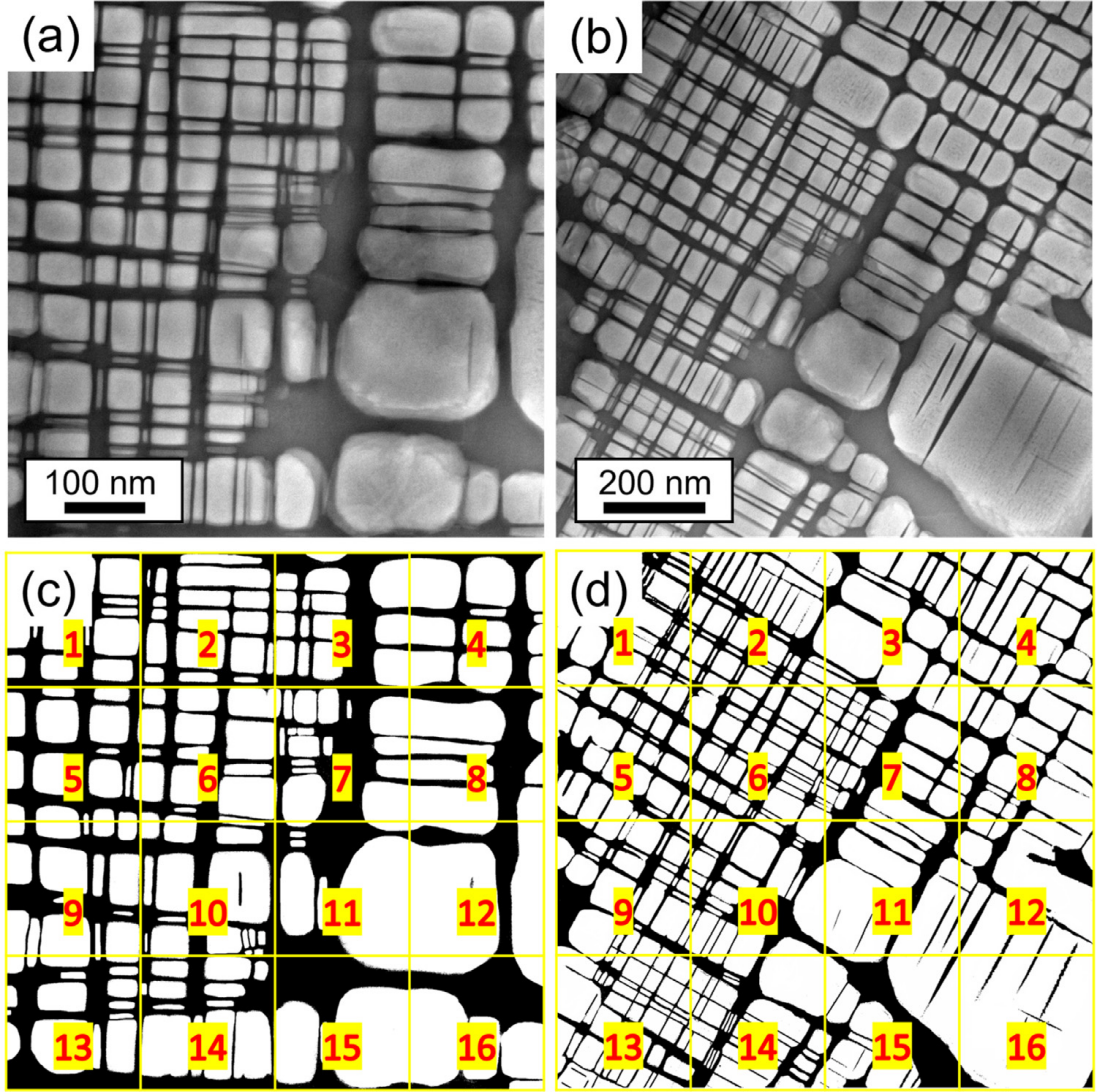
(a,b) STEM-HAADF micrographs of the AN alloy used for the area fraction determination at (a) 200kx and (b) 120kx. (b,c) Binarized images of (a) and (b), respectively. The black phase shown in (c,d) represents the B2 phase (channels). The binarized images were divided into 16 images, as shown by the yellow squares and red numbers.

The area fractions of the Al-Zr-rich phase were determined for both states using BSE images and the B2/A2 phases using STEM-HAADF images with the image analysis software (ImageJ) [3] (Table 3). Four BSE images were used to determine the area fraction of the Al-Zr-rich phase in both states. The images were converted to binary images and separated into two different phases using the “threshold” tool of ImageJ. After this step, the binary image was divided into 16 images, which were analyzed separately. Fig. 4 (AC) and Fig. 5 (AN) show the images used to determine the area fraction (left), with the respective binary image separating the Al-Zr-rich phase from the rest of the area (right, black phase on white background). Table 7 (AC) and Table 8 (AN) show the percentage area of the Al-Zr-rich phase for each analyzed image in Fig. 4 and Fig. 5, respectively. For the determination of the area fraction of the B2 phase, two images were used for the AC alloy (Fig. 6 and Fig. 5d in Ref. [1]) and three images were used for the AN alloy (Fig. 7 and Fig. A3a in the supplementary material of Ref. [1]).

**Table 7:** Area fraction of the Al-Zr-rich phase in the AC state, determined from the micrographs in Fig. 4, using ImageJ. The uncertainty is given by the standard deviation.

	Fig. 4e	Fig. 4f	Fig. 4g	Fig. 4h
	
	
Sub-images in the binarized images (shown in Fig. 4e-h)	% Area Al-Zr phase (AC alloy)			
1	11.1	6.5	7.6	9.5
2	6.2	10.4	6.7	8.1
3	8.6	7.5	6.6	8.9
4	6.9	8.2	6.8	8.6
5	8.1	5.8	6.9	4.9
6	17.0	5.7	7.5	10.2
7	4.1	7.0	9.1	6.0
8	1.4	7.7	10.2	5.8
9	15.1	7.2	8.2	11.7
10	17.1	9.0	5.8	8.1
11	14.1	6.2	5.7	8.2
12	5.2	7.5	6.2	7.0
13	15.2	7.7	8.1	9.5
14	18.7	7.1	5.8	5.3
15	2.7	8.4	5.3	3.7
16	19.4	9.3	11.3	7.0

**Table 8:** Area fraction of the Al-Zr-rich phase in the AN state, determined from the micrographs in Fig. 5, using ImageJ. The uncertainty is given by the standard deviation. .

	Fig. 5e	Fig. 5f	Fig. 5g	Fig. 5h
	
	
Sub-images in the binarized images (shown in Fig. 5e-h)	% Area Al-Zr phase (AN alloy)			
1	8.6	17.5	13.5	10.4
2	11.6	8.9	13.2	11.3
3	12.2	12.5	24.7	9.8
4	12.0	9.7	21.7	5.7
5	9.0	12.2	17.3	9.9
6	6.9	10.3	11.0	12.6
7	9.4	12.8	22.4	12.3
8	12.4	8.1	30.9	12.5
9	9.0	10.3	15.5	14.6
10	7.4	7.2	19.8	16.3
11	8.5	9.7	23.4	8.5
12	9.4	6.6	31.7	8.6
13	5.3	17.6	14.0	11.4
14	5.9	10.6	28.9	11.9
15	5.1	11.2	18.2	14.8
16	10.8	11.0	22.3	6.9

Fig. 6 (AC) and Fig. 7 (AN) also show the corresponding binary images in which the B2 phase channels (black) are separated from the rest of the region (white background). Table 9 (AC) and Table 10 (AN) show the area fraction in% of the B2 phase for each analyzed image in Fig. 6 and Fig. 7, respectively. The thickness of the B2 channels and A2 plates were measured manually ≈ 200 times using the ImageJ program.

**Table 9:** Area fraction (%) of the B2 phase in the AC state, determined from Fig. 6b and c, using ImageJ software. The uncertainty is given by the standard deviation.

	Fig. 6b	Fig. 6c
	
	
Sub-images in the binarized images (shown in Fig. 6b and c)	Area fraction of B2 phase in AC alloy (%)	
1	38.0	42.6
2	35.6	53.3
3	32.9	41.8
4	30.4	40.8
5	38.6	43.2
6	37.0	40.7
7	30.7	38.9
8	32.6	40.7
9	38.0	40.7
10	39.7	42.0
11	34.5	40.4
12	33.4	39.6
13	39.9	42.4
14	39.2	41.2
15	34.9	37.9
16	34.7	41.6

**Table 10:** Area fraction (%) of the B2 phase in the AN state, determined from Fig. 7c and d, using the program ImageJ. The uncertainty is given by the standard deviation.

	Fig. 7c	Fig. 7d
	
	
Sub-images in the binarized images (shown in Fig. 7c and d)	Area fraction of B2 phase in AN alloy (%)	
1	45.1	40.8
2	32.9	42.7
3	25.1	43.9
4	23.8	42.7
5	36.5	37.5
6	29.8	40.1
7	35.5	63.6
8	22.1	48.4
9	40.5	42.4
10	28.9	42.4
11	24.8	36.4
12	21.9	25.6
13	12.3	57.6
14	10.9	40.1
15	18.1	33.7
16	4.2	33.7

**Fig. 8:**
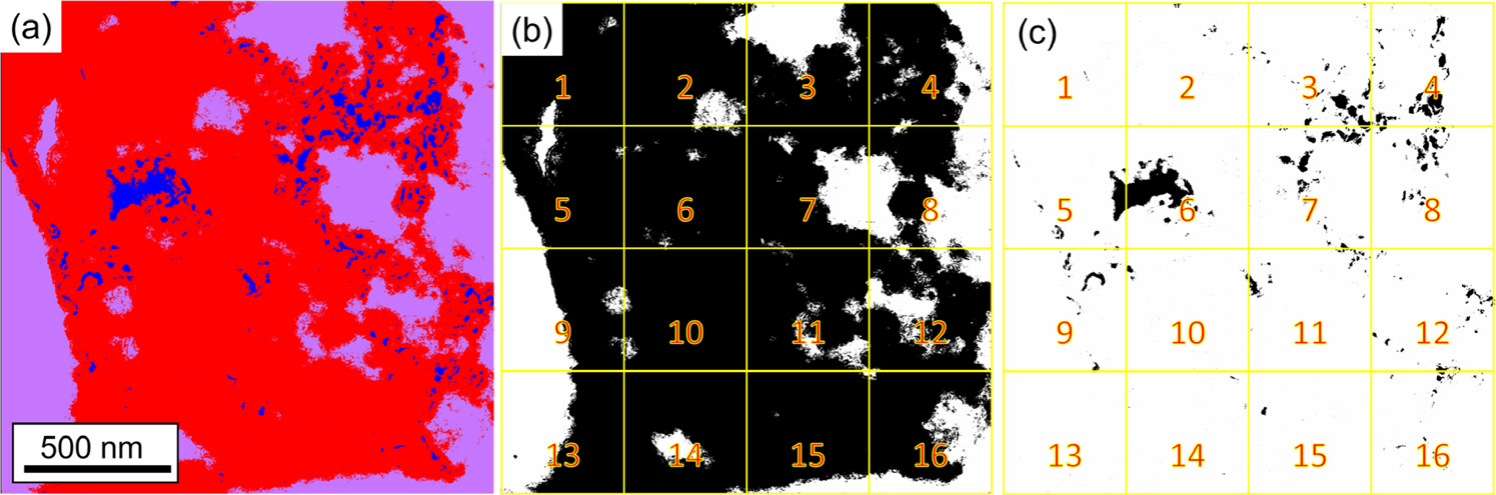
Determination of the percentage (%) of the amorphous phase in the Al-Zr-rich phase (a) separation of the three different phases using the software´s “Trainable Weka Segmentation” tool (b) binary image considering the fully Al-Zr-rich phase region (c) binary image only considering the crystal phase in the Al-Zr-rich region.

Fig. 8 shows the procedure undertaken to determine the area fraction of the amorphous Al-Zr-rich phase for the original CTEM-DF micrograph in Fig. 6b of Ref. [1]. The tool “Trainable Weka Segmentation”, embedded in the FIJI of ImageJ [3], allows the separation of three different regions of interest for their area fraction measurement, as shown in Fig. 6b in Ref. [1] (crystalline (bright regions), amorphous (light gray regions) Al-Zr-rich phase, A2/B2 regions (dark)). In Fig. 8a, the red color is the amorphous Al-Zr-rich phase (cf. light gray in Fig. 6b of Ref. [1]), the blue region is the crystalline region (cf. bright regions in Fig. 6b of Ref. [1]), and the lilac region is the surrounding thick A2/B2 in the micrograph (cf. dark regions in Fig. 6b of Ref. [1]).

Fig. 8a is then binarized (Fig. 8b), where the entire black region is the Al-Zr-rich phase (including the amorphous and crystalline structure, i.e., red plus blue in Fig. 8a), and the “white region” is the rest of the sample (i.e., thick A2/B2 region in lilac, Fig. 8a). Fig. 8b is divided into 16 sub-images to analyze their area fraction separately. Finally, Fig. 8a is binarized again (Fig. 8c), but now considering only the crystalline region (blue phase in Fig. 8a) and divided into 16 images. Table 11 presents the area fraction of the total Al-Zr-rich phase (second column, from the black phase in Fig. 8b) and the crystalline phase (third column, from the black phase in Fig. 8c). Weighing the area fraction of the crystalline phase relative to the total Al-Zr-rich phase, the fourth column in Table 11 gives the amount of crystalline phase embedded in the Al-Zr-rich phase.

**Table 11:** Area fraction of the Al-Zr-rich phase and crystalline phase in the Al-Zr-rich phase determined from the images in Fig. 8a–c.

Sub-images from Fig. 8b,c	Area fraction only Al-Zr-phase (Fig. 8b)	Area fraction only crystalline phase (Fig. 8c)	Area fraction of crystalline phase
1	98.0	0.6	0.6
2	90.8	0.9	1.0
3	65.2	5.3	8.1
4	68.8	7.6	11.1
5	79.6	2.6	3.3
6	97.2	14.4	14.8
7	76.4	7.5	9.8
8	44.4	5.3	11.9
9	46.4	3.0	6.5
10	97.3	1.7	1.8
11	90.3	2.0	2.2
12	72.7	2.9	4.0
13	44.7	0.3	0.7
14	93.8	0.5	0.5
15	90.6	0.7	0.8
16	64.1	1.9	3.0
Average	76.3 ± 19.2	3.6 ± 3.7	5.0 ± 4.7

The average chemical compositions of the phases listed in Table 2 of Ref. [1] were determined using energy dispersive spectroscopy (EDX) in a Quanta 3D SEM with an EDAX Octane Elect SDDs detector at 30 kV (WD 10 mm) and in a JEOL JEM-2200FS TEM with an EDX Jeol JED-23000BU Si (Li) detector with ultrathin organic/Al window at 200 kV.

The Al-Zr-rich phase composition was determined from the average of the analyzes of two regions with TEM-EDX measurements for each state (AC and AN). The dendritic and interdendritic compositions were determined from the average of five spot analyzes (SEM-EDX) for the AC in the electron-transparent regions (thickness < 1 µm) of a TEM specimen.

The chemical compositions of the A2 and B2 phases were determined from the average of three spot analyzes (TEM-EDX) for the AN specimen, and finally, the chemical composition of the bright phase surrounding the Al-Zr-rich phase was determined using the average of three spot analyzes (SEM-EDX) in the AN alloy.

Name, description and format of each dataset files hosted in Mendeley Repository.

**Table:** 

Folder	File name	Description	Format
XRD	Al4Zr5_00-048-1382.cif	Diffraction data of the Al_4_Zr_5_ phase used to simulate the diffraction pattern (.cif file)	Raw
	AlTiZr_Struktur_annealing.cel	Simulated diffraction pattern of the B2 phase after annealing obtained with Powdercell software	Analyzed
	AlTiZr_Struktur_as-cast.cel	Simulated diffraction pattern of the B2 phase after casting obtained with Powdercell software	Analyzed
	AlZr_Hexagonal.cel	Simulated diffraction pattern of the Al-Zr-rich phase after casting obtained with Powdercell software	Analyzed
	MoNbTa_Struktur_annealing.cel	Simulated diffraction pattern of the A2 phase after annealing obtained with Powdercell software	Analyzed
	MoNbTa_Struktur_as-cast.cel	Simulated diffraction pattern of the B2 phase after casting obtained with Powdercell software	Analyzed
	XRDPattern_annealed_10–120.xlsx	Experimental XRD pattern of the sample in annealed state	Raw
	XRDPattern_as-cast_10–100.xlsx	Experimental XRD pattern of the sample in cast state	Raw

SEM	Micrograph_grainsize_AC_BSE_01.tif	SEM-BSE micrograph N° 1 used to get the grain size in cast state	Raw
	Micrograph_grainsize_AC_BSE_02.tif	SEM-BSE micrograph N° 2 used to get the grain size in cast state	Raw
	Micrograph_grainsize_AC_BSE_03.tif	SEM-BSE micrograph N° 3 used to get the grain size in cast state	Raw
	Micrograph_grainsize_AC_BSE_04.tif	SEM-BSE micrograph N° 4 used to get the grain size in cast state	Raw
	Micrograph_grainsize_AN_BSE_01.tif	SEM-BSE micrograph N° 1 used to get the grain size in annelaed state	Raw
	Micrograph_grainsize_AN_BSE_02.tif	SEM-BSE micrograph N° 2 used to get the grain size in annelaed state	Raw
	Micrograph_grainsize_AN_BSE_03.tif	SEM-BSE micrograph N° 3 used to get the grain size in annelaed state	Raw
	Micrograph_grainsize_AN_BSE_04.tif	SEM-BSE micrograph N° 4 used to get the grain size in annelaed state	Raw
	BSE_AC_01_processed.tif	SEM-BSE micrograph N° 1 used to get the volume fraction of the Al-Zr-rich phase in cast state	Raw
	BSE_AC_01_processed_Threshold.tif	Threshold processed SEM-BSE micrograph N° 1 used to get the volume fraction of the Al-Zr-rich phase in cast state	Analyzed
	BSE_AC_01_processed_Threshold_16images.cvs	Excel file that contains the% of area fraction of the Al-Zr-rich phase in the cast state, after division of the Threshold image into 16 fields of the SEM-BSE micrograph N° 1	Analyzed
	BSE_AC_02_processed_processed.tif	SEM-BSE micrograph N° 2 used to get the volume fraction of the Al-Zr-rich phase in cast state	Raw
	BSE_AC_02_processed_Threshold.tif	Threshold processed SEM-BSE micrograph N° 2 used to get the volume fraction of the Al-Zr-rich phase in cast state	Analyzed
	BSE_AC_02_processed_Threshold_16images.cvs	Excel file that contains the% of area fraction of the Al-Zr-rich phase in the cast state, after division of the Threshold image into 16 fields of the SEM-BSE micrograph N° 2	Analyzed
	BSE_AC_03_processed.tif	SEM-BSE micrograph N° 3 used to get the volume fraction of the Al-Zr-rich phase in cast state	Raw
	BSE_AC_03_processed_Threshold.tif	Threshold processed SEM-BSE micrograph N° 3 used to get the volume fraction of the Al-Zr-rich phase in cast state	Analyzed
	BSE_AC_03_processed_Threshold_16images.cvs	Excel file that contains the% of area fraction of the Al-Zr-rich phase in the cast state, after division of the Threshold image into 16 fields of the SEM-BSE micrograph N° 3	Analyzed
	BSE_AC_04_processed.tif	SEM-BSE micrograph N° 4 used to get the volume fraction of the Al-Zr-rich phase in cast state	Raw
	BSE_AC_04_processed_Threshold.tif	Threshold processed SEM-BSE micrograph N° 4 used to get the volume fraction of the Al-Zr-rich phase in cast state	Analyzed
	BSE_AC_04_processed_Threshold_16images.csv	Excel file that contains the% of area fraction of the Al-Zr-rich phase in the as-cast state, after division of the Threshold image into 16 fields of the SEM-BSE micrograph N° 4	Analyzed
	BSE_AN_01_processed_Threshold.tif	Threshold processed SEM-BSE micrograph N° 1 used to get the volume fraction of the Al-Zr-rich phase in annealed state	Analyzed
	BSE_AN_01_processed_Threshold_16images.csv	Excel file that contains the% of area fraction of the Al-Zr-rich phase in annealed state, after division of the Threshold image into 16 fields of the SEM-BSE micrograph N° 1	Analyzed
	BSE_AN_02_processed_Threshold.tif	Threshold processed SEM-BSE micrograph N° 2 used to get the volume fraction of the Al-Zr-rich phase in annealed state	Analyzed
	BSE_AN_02_processed_Threshold_16images.csv	Excel file that contains the% of area fraction of the Al-Zr-rich phase in annealed state, after division of the Threshold image into 16 fields of the SEM-BSE micrograph N° 2	Analyzed
	BSE_AN_03_processed_Threshold.tif	Threshold processed SEM-BSE micrograph N° 3 used to get the volume fraction of the Al-Zr-rich phase in annealed state	Analyzed
	BSE_AN_03_processed_Threshold_16images.csv	Excel file that contains the% of area fraction of the Al-Zr-rich phase in annealed state, after division of the Threshold image into 16 fields of the SEM-BSE micrograph N° 3	Analyzed
	BSE_AN_04_processed_Threshold.tif	Threshold processed SEM-BSE micrograph N° 4 used to get the volume fraction of the Al-Zr-rich phase in annealed state	Analyzed
	BSE_AN_04_processed_Threshold_16images.csv	Excel file that contains the% of area fraction of the Al-Zr-rich phase in annealed state, after division of the Threshold image into 16 fields of the SEM-BSE micrograph N° 4.	Analyzed

TEM	CTEM-DF_AC_Al-Zr-phase.dm3	CTEM-DF micrograph in the Al-Zr-rich region (Inluding amorphous and crystalline regions) of the sample in the cast state. Fig. 6b of Ref. [1]	Raw
	CTEM-BF_AC_interdendritic_001zoneaxis_a.dm3	CTEM-BF micrograph in the interdendritic region orineted along the 001 zone axis of the sample in the cast state Fig. 3a)	Raw
	SADP_AC_interdendritic_001zoneaxis_b.dm3	Selected area diffraction pattern (SADP) in the interdendritic region oriented along the 001 zone axis of the sample in the cast state (Fig. 3b)	Raw
	CTEM-DF_AC_Al-Zr-Classified image.tif	CTEM-DF micrograph of the sample in the cast state in the Al-Zr-rich region with separation of the three different phases (surrounding, Al-Zr-rich amorphous phase, and Al-Zr-rich crystallline phase) using the software´s “Trainable Weka Segmentation” tool of FIJI (Fig. 8a of present work)	Analyzed
	CTEM-DF_AC_Al-Zr_amorphous_only_bin.tif	Binary image of the CTEM-DF micrograph of the sample in the cast state in the Al-Zr-rich region considering the fully Al-Zr-rich phase region and the surrounding (Fig. 8b)	Analyzed
	CTEM-DF_AC_Al-Zr_amorphous_only_bin.csv	Excel file that contains the% of area fraction of the complete area of Al-Zr-rich phase in the cast state (Fig. 8b)	Analyzed
	CTEM-DF_AC_Al-Zr_Crystals.tif	Binary image of the CTEM-DF micrograph of the sample in the Al-Zr-rich region only considering the crystal phase in the cast state (Fig. 8c)	Analyzed
	CTEM-DF_AC_Al-Zr_crystals.csv	Excel file that contains the% of area fraction of the crystalline area (Al-Zr-rich phase) in the cast state (Fig. 8c)	Analyzed
	STEM-HAADF_AC_bcc-B2areafraction.dm3	STEM-HAADF micrograph N° 1 of the AC alloy used to determine the area fraction of A2/B2 phases (Fig. 6a)	Raw
	STEM-HAADF_AC_bcc-B2fraction_Bandpassfilter.tif	STEM-HAADF micrograph N° 1 of the AC alloy used to determine the area fraction of A2/B2 phases with Bandpass filter applied with FIJI software	Analyzed
	STEM-HAADF_AC_bcc-B2fraction_T_16images.tif	Threshold separation of the STEM-HAADF micrograph of the AC alloy used to determine the area fraction of A2/B2 phases with Bandpass filter applied with FIJI software (Fig. 6b)	Analyzed
	STEM-HAADF_AC_bcc-B2fraction_T_16images.cvs	Excel file that contains the% of area fraction of the A2/B2 phases in the cast state of the micrograph N° 1	Analyzed
	STEM-HAADF_AC_bcc-B2fraction_2.dm3	STEM-HAADF micrograph N° 2 of the AC alloy used to determine the area fraction of A2/B2 phases (Fig. 5d of Ref. [1])	Raw
	STEM-HAADF_AC_bcc-B2_2_Bandpassfilter.tif	STEM-HAADF micrograph N° 2 of the AC alloy used to determine the area fraction of A2/B2 phases with Bandpass filter applied with FIJI software	Analyzed
	STEM-HAADF_AC_bcc-B2_2_Bandpassfil_16images.tif	Threshold separation of the STEM-HAADF micrograph of the AC alloy used to determine the area fraction of A2/B2 phases with Bandpass filter applied with FIJI software (Fig. 6c)	Analyzed
	STEM-HAADF_AC_bcc-B2_2_Bandpassfilter_16ima.cvs	Excel file that contains the% of area fraction of the A2/B2 phases in the cast state of the micrograph N° 2	Analyzed
	STEM-HAADF_AN_bcc-B2fraction_01.dm3	STEM-HAADF micrograph N° 1 of the AN alloy used to determine the area fraction of A2/B2 phases (Fig. 6a)	Raw
	STEM-HAADF_AN_bcc-B2fraction_01_threshold_16images.jpg	Threshold separation of the STEM-HAADF micrograph N° 1 of the AC alloy used to determine the area fraction of A2/B2 phases with Bandpass filter applied with FIJI software (Fig. 6b)	Analyzed
	STEM-HAADF_AN_bcc-B2fraction_01_threshold.cvs	Excel file that contains the% of area fraction of the A2/B2 phases in the annealed state of the micrograph N° 1	Analyzed
	STEM-HAADF_AN_bcc-B2fraction_02.dm3	STEM-HAADF micrograph N° 2 of the AN alloy used to determine the area fraction of A2/B2 phases (Fig. 6a)	Raw
	STEM-HAADF_AN_bcc-B2fraction_02_threshold_16images.tif	Threshold separation of the STEM-HAADF micrograph N° 2 of the AN alloy used to determine the area fraction of A2/B2 phases with Bandpass filter applied with FIJI software (Fig. 6b)	Analyzed
	STEM-HAADF_AN_bcc-B2fraction_02_threshold_.cvs	Excel file that contains the% of area fraction of the A2/B2 phases in the annealed state of the micrograph N° 2	Analyzed
	precipitates_as-cast.cvs	Excel file that contains the length of the A2 phase (precipitates) in the annealed state	Analyzed
	precipitates_annealed.cvs	Excel file that contains the length of the A2 phase (precipitates) in the cast state	Analyzed
	channels_annealed.cvs	Excel file that contains the length of the B2 phase (channels) in the annealed state	Analyzed
	channels_as-cast.cvs	Excel file that contains the length of the B2 phase (channels) in the cast state	Analyzed

EDX	Mapping_Annealing	This folder contains the raw data of the EDX elemental map for the AN sample shown in Fig. A1 in supplemetary data of Ref. [1].	Raw
	Mapping_As-cast	This folder contains the raw data of the EDX elemental map for the AC sample shown in Fig. 3 of Ref. [1].	Raw
	EDX-SEM_AC_Al-Zr-phase_01.docx	EDX Point analysis used to determine the average chemical composition of the Al-Zr-rich phase in the AC sample using SEM	Raw
	EDX-SEM_AC_dendritic-interdendritic_01.docx	EDX Point analysis used to determine the average chemical composition of the dendritic and interdendritic regions of the AC sample	Raw
	EDX-TEM_AC_Al-Zr-phase_02.ppt	EDX area analysis used to determine the average chemical composition of the Al-Zr-rich phase in the AC sample using TEM	Raw
	EDX-TEM_AN_Al-Zr-phase.dm3	STEM micrpgraph of the region used to determine the chemical composition of the Al-Zr-rich phase in the AN sample using EDX-TEM	Raw
	EDX-TEM_AN_Al-Zr-phase.ppt	EDX area analysis used to determine the average chemical composition of the Al-Zr-rich phase in the AN sample using TEM	Raw
	EDX-TEM_AN_bcc-B2-phase.dm3	STEM micrpgraph of the region used to determine the chemical composition of the A2 and B2 phases in the AN sample using EDX-TEM	Raw
	EDX-TEM_AN_bcc-B2-phase.ppt	EDX point analysis used to determine the average chemical composition of the A2 and B2 phases in the AN sample using TEM	Raw

## Ethics Statements

This work does not require any ethical statement.

## CRediT authorship contribution statement

**Patricia Suárez Ocaño:** Methodology, Software, Data curation, Writing – original draft. **Leonardo Agudo Jácome:** Conceptualization, Methodology, Writing – review & editing, Supervision, Funding acquisition. **Inmaculada Lopez-Galilea:** Writing – review & editing. **Reza Darvishi Kamachali:** Writing – review & editing. **Suzana G. Fries:** Writing – review & editing.

## Declaration of Competing Interest

The authors declare that they have no known competing financial interests or personal relationships that could have appeared to influence the work reported in this paper.

## Data Availability

Data regarding the AlMo0.5NbTa0.5TiZr refractory high entropy superalloy (Original data) (Mendeley Data).
